# Legionnaire’s Disease Presenting With the Legionella Triad (Pneumonia, Rhabdomyolysis, and Renal Failure) and Cardiac Complications

**DOI:** 10.7759/cureus.26056

**Published:** 2022-06-18

**Authors:** Arjun Prasanna, Justin Palmer, Sharon Wang

**Affiliations:** 1 Infectious Disease, California University of Science and Medicine, Colton, USA; 2 Internal Medicine, Arrowhead Regional Medical Center, Colton, USA; 3 Infectious Disease, Arrowhead Regional Medical Center, Colton, USA

**Keywords:** infectious disease medicine, pulmonary critical care, legionnaires disease, new onset atrial fibrillation, non-st segment elevation myocardial infarction (nstemi), rhabdomyolysis with acute renal failure, legionella pneumonia

## Abstract

*Legionella* pneumonia is well-characterized as a cause of atypical, community-acquired pneumonia in susceptible individuals. In recent years, extrapulmonary manifestations, including an emerging triad of *Legionella* pneumonia, rhabdomyolysis, and renal failure, have been identified. Here we report a case of Legionnaire's disease that presented non-classically, with subclinical pneumonia and non-ST elevated myocardial infarction (NSTEMI). Although he received early treatment with antibiotics, he developed the *Legionella*triad during admission. He had several episodes of cardiopulmonary decompensation during his hospital course, eventually ending in his passing. This case serves to highlight the importance of early identification and intervention in regard to extrapulmonary *Legionella* infection.

## Introduction

Legionellosis, caused by the pathogen *Legionella*, varies in manifestation from mild to severe disease based on the patient's risk factors. *Legionella* species are natively found in both natural and artificial water systems such as hot tubs, pools, saunas, and, in this case, a home water fountain [1]. Patients diagnosed with legionellosis are commonly susceptible individuals that are exposed to an aerosolized aquatic environment. Other significant risk factors include a smoking history and an immunocompromised state. This case report illustrates a case of an immunocompromised individual who, while initially improved with antibiotics, unfortunately, expired after an episode of cardiopulmonary arrest.

## Case presentation

A 79-year-old man with a chronic myeloproliferative disorder (on ruxolitinib), hypertension (HTN), and type 2 diabetes mellitus (DM) presented to the emergency department (ED) after a fall from a poorly assembled chair without loss of consciousness (LOC). His social history and review of systems were unremarkable.

In the ED, the patient was found to be febrile (100.9 °F) and the rest of his vitals were within normal limits, including oxygen saturation (SpO2) at 100%. His labs were significant for moderate leukocytosis (although unknown baseline given hematologic disorder), moderate hyponatremia of 125 mEq/L, serial elevated troponins, creatinine (Cr) of 1.7 mg/dL with unknown baseline, azotemia, and elevated lactate (Table 1). An electrocardiogram (EKG) showed a right bundle branch block and nonspecific T-wave changes. His CURB-65 (confusion, urea nitrogen, respiratory rate, blood pressure, age$\geq$65 years) score was 2 given his age and azotemia. A chest radiograph showed bilateral consolidation suggestive of multifocal pneumonia. Ceftriaxone and azithromycin were initiated in addition to heparin drip. He was admitted for pneumonia and NSTEMI.

**Table 1:** Laboratory analysis at admission WBC, white blood cells; Hgb, hemoglobin; Na, sodium; K, potassium; BUN, blood urea nitrogen; LDH, lactate dehydrogenase

Laboratory analysis	Level	Normal Range
WBC	20.3 k/uL	4.3-11.1 k/uL
Segmented neutrophils	87%	55-70%
Band neutrophils	9%	2-5%
Leukocytes	2%	20-48%
Hgb	11.1 g/dL	13-17 g/dL
Platelets	255 k/uL	120-360 k/uL
Na	125 mEq/L	135-145 mEq/L
K	5.4 mEq/L	3.5-5.5 mEq/L
BUN	36 mg/dL	7-20 mg/dL
Creatinine	1.7 mg/dL	0.5-1.5 mg/dL
Rand Glucose	485 mg/dL	65-125 mg/dL
Lactate	4.54 mmol/L	0.5-2.0 mmol/L
LDH	448 u/L	120-230 u/L
Creatine Kinase	265 u/L	30-170 u/L
Troponin I	0.42, 2.11, 3.9 ng/mL	0-0.30 ng/mL

On Day 1 of hospitalization, the patient was hypoxic on 2L oxygen via nasal cannula (NC) saturating at 72% and tachypneic using accessory muscles to breathe, hypertensive (BP 190/91), and tachycardic. Chest radiograph showed worsening consolidation and airspace disease. EKG was unchanged. He was started on a high-flow nasal cannula (HFNC) with 45% FiO2 with a 70L flow rate. He was also given labetalol for BP and HR control. Echocardiography showed grade II diastolic heart failure with an ejection fraction of 45%-50%, and the patient was started on aspirin (ASA) 81 mg and atorvastatin 40 mg. IV antibiotics were escalated to cefepime and vancomycin. He was upgraded to the intensive care unit given his progressive acute hypoxemic respiratory failure.

**Figure 1:**
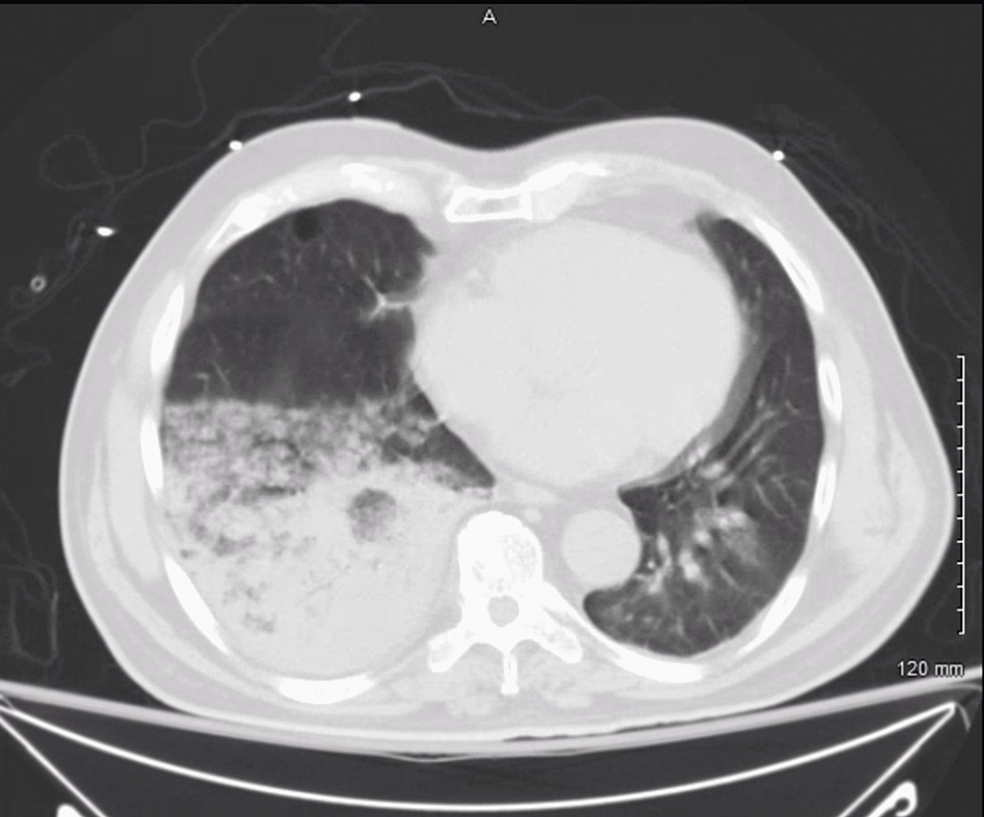
Axial CT scan showing right lower lobe consolidation

On hospital Day 2, the patient's respiratory status continued to decline, now requiring bilevel positive airway pressure (BiPAP). A chest computerized tomography (CT) scan confirmed multifocal pneumonia with right lower lobe consolidation (Figure 1). An EKG now showed new-onset atrial fibrillation with a rapid ventricular response (Afib with RVR), with a heart rate in the 160s. The rate was controlled with diltiazem, metoprolol, and digoxin drip. Given his unresolving acute kidney injury (AKI), creatine kinase was ordered and found to be elevated at 3802 U/L, increased from 265 U/L at admission, concerning for acute tubular necrosis.

**Figure 2:**
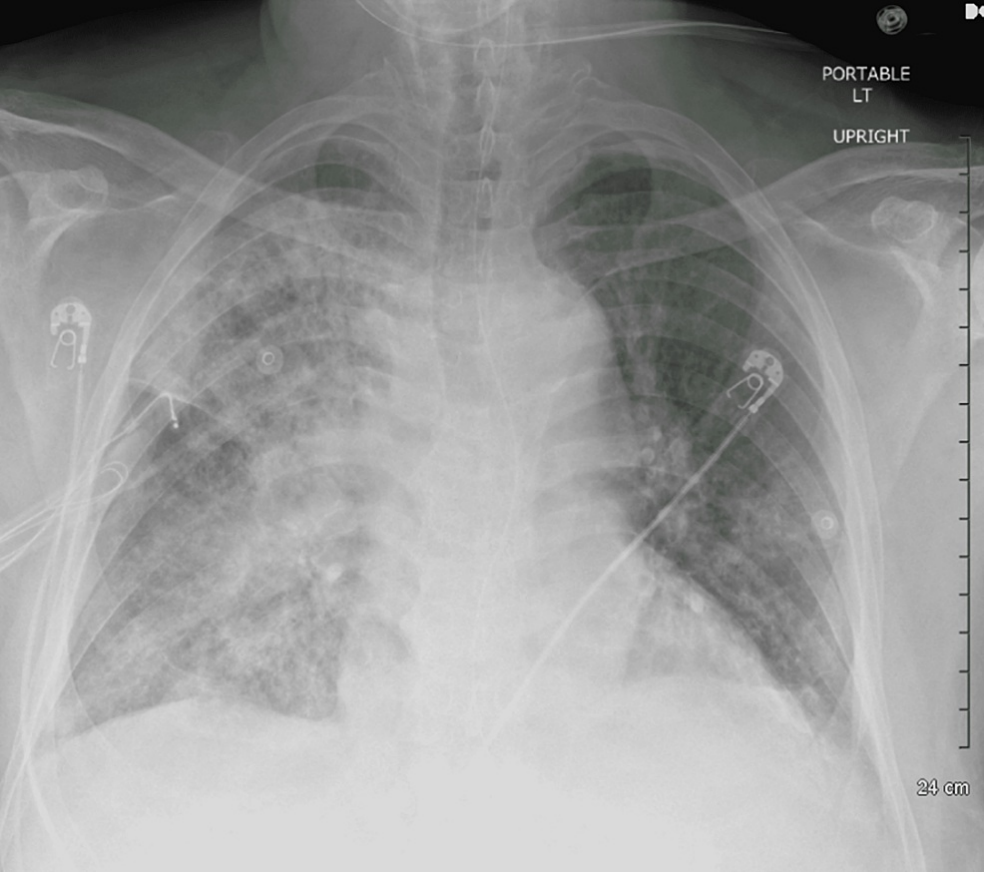
Chest X-ray showing diffuse right lung consolidation

On Day 4 of hospitalization, the patient's diagnosis of legionellosis was confirmed with a positive *Legionella* urine antigen test. Of note, the patient endorsed that in the days prior to admission, he had worked outside in his home garden and was engaged in the maintenance of a water fountain. The patient was continued on azithromycin, and antibiotics were further adjusted to ampicillin/sulbactam for coverage of other organisms not exclusive to* Legionella*. Chest radiograph demonstrated persistent multifocal pneumonia with right lung consolidation (Figure 2).

On Day 5 of hospitalization, the patient demonstrated improved respiratory status, weaning oxygen requirements from HFNC to NC and intermittently to room air (RA). He was downgraded to the telemetry unit and remained hemodynamically stable, with evidence of renal recovery as well.

On Day 9 of hospitalization, the patient was again found tachypneic, hypotensive (97/65), and tachycardic but without hypoxia. Hemoglobin was found to be 4.5 g/dL. Of note, his platelet count had remained stable to this point from admission. A massive transfusion protocol and further workup were offered but declined by the family. The patient passed away on Day 9.

## Discussion

Legionnaire's disease is classically known to present with a constellation of findings, including pneumonia, diarrhea, and hyponatremia. More recently, a rare triad of pneumonia, rhabdomyolysis, and acute renal failure has been identified in patients with Legionnaires disease and is associated with high morbidity and mortality [2-6]. The exact pathophysiology of rhabdomyolysis and renal failure in the setting of *Legionella* infection is poorly understood and is currently suspected to be endotoxin mediated. The two predominant theories are thought to be rhabdomyolysis-induced versus direct bacterial inoculation of renal tissue as supported by biopsy-proven cases [5]. Despite an initially asymptomatic course, our patient eventually developed all three components of the *Legionella* triad.

Although our patient’s myocardial ischemia was presumed to be due to hypoperfusion in the setting of sepsis and possible underlying coronary disease, a full ischemic workup was not completed. Recent case reports demonstrate an association of *Legionella* pneumonia with extrapulmonary manifestations such as endocarditis, myocarditis, and pericarditis [7]. There are also reports of *Legionella* myocarditis with pathology-confirmed myocardial biopsies [8]. Disseminated *Legionella* infection with myocardial inoculation was unlikely to be the sole cause but possibly a minor contributing factor in this patient's cardiovascular complications. In addition, this patient's new-onset A-Fib with RVR was likely secondary to ischemic conduction pathway damage in the setting of his NSTEMI on admission.

The exact cause of our patient's demise was undetermined, although presumed to be secondary to a massive gastrointestinal or intra-abdominal hemorrhage in the setting of progressive acute respiratory failure and multiorgan decline. It is possible that acute ruxolitinib toxicity may have contributed to his drop in hemoglobin given his recent acute renal injury and use of diltiazem (a CYP34A inhibitor).

Diagnostic considerations

A thorough history and physical can often hint at legionellosis sooner than diagnostic tests. Information about an immunocompromised state, smoking history, and social history involving exposure to aerosolized water is therefore important to obtain. The Centers for Disease Control (CDC) currently recommends obtaining a respiratory culture if possible, as this is most sensitive for all *Legionella *serotypes. Urinary antigen testing is also an excellent test that provides a rapid, same-day result unaffected by antibiotics [9-10]. Urinary antigen testing can only be used to detect *Legionella pneumophilia* serogroup 1 (Lp1), however, Lp1 may account for up to 84% of all cases of *Legionella* pneumonia. Furthermore, polymerase chain reaction (PCR) testing for *Legionella* antigen has recently been Federal Drug Administration (FDA) approved for diagnostic use. It is highly sensitive and specific for several serogroups, including Lp1 [11]. There are other methods, such as serologic testing and immunofluorescence, however, these are not the first line due to challenges in obtaining the appropriate specimens [10].

Treatment

Empiric coverage for community-acquired pneumonia in hospitalized adults often overlaps with the most current treatment for* Legionella* pneumonia. The current antibiotic strategy entails a seven to 10-day course of monotherapy with a macrolide or respiratory fluoroquinolone, particularly azithromycin or levofloxacin, respectively [12]. Combination therapy with a macrolide plus fluoroquinolone has not been shown to be any more effective than monotherapy, and therefore whichever antibiotic is better tolerated and/or provides coverage for concomitant infections should be selected [13-15]. Although most studies agree that there is no significant difference in mortality benefit between macrolides versus fluoroquinolone monotherapy, there is still some debate about whether there is a clinically significant reduction in length of hospital stay associated with fluoroquinolone monotherapy in comparison to macrolide monotherapy [15-16].

## Conclusions

*Legionella* infection can be complicated by a rare triad of pneumonia, rhabdomyolysis, and acute kidney injury. Emerging case reports are providing further evidence of extrapulmonary manifestations, including cardiovascular inoculation. The *Legionella* triad is rare and appears to be associated with a worse prognosis than *Legionella* pneumonia alone, particularly with cardiac involvement. A thorough history and workup are essential to begin treatment promptly and prevent acute decompensation with multiorgan failure.

## References

[1] Cunha BA, Burillo A, Bouza E (2016). Legionnaires’ disease. Lancet.

[2] Kassha K, Abuanza I, Hadi SA, Hilton R (2009). Severe Legionnaires disease complicated by multi-organ dysfunction in a previously healthy patient: a case report. Cases J.

[3] Seegobin K, Maharaj S, Baldeo C, Downes JP, Reddy P (2017). Legionnaires’ disease complicated with rhabdomyolysis and acute kidney injury in an AIDS patient. Case Rep Infect Dis.

[4] Sutarjono B, Alexis J, Sachidanandam JC (2019). Legionella pneumonia complicated by rhabdomyolysis. BMJ Case Rep.

[5] Soni AJ, Peter A (2019). Established association of legionella with rhabdomyolysis and renal failure: a review of the literature. Respir Med Case Rep.

[6] Erdogan H, Yilmaz A, Kal O, Erdogan A, Arslan H (2006). Rhabdomyolysis-induced acute renal failure associated with Legionnaires' disease. Scand J Urol Nephrol.

[7] Schürmann D, Grosse G, Horbach I, Fehrenbach FJ (1983). Pulmonary and extrapulmonary manifestations of L. pneumophila. Zentralbl Bakteriol Mikrobiol Hyg A.

[8] Ishimaru N, Suzuki H, Tokuda Y, Takano T (2012). Severe Legionnaires' disease with pneumonia and biopsy-confirmed myocarditis most likely caused by Legionella pneumophila serogroup 6. Intern Med.

[9] Sharma L, Losier A, Tolbert T, Dela Cruz CS, Marion CR (2017). Atypical pneumonia: updates on Legionella, Chlamydophila, and Mycoplasma pneumonia. Clin Chest Med.

[10] (2022). Legionnaires disease diagnosis, treatment, and prevention. CDC.

[11] Avni T, Bieber A, Green H, Steinmetz T, Leibovici L, Paul M (2016). Diagnostic accuracy of PCR alone and compared to urinary antigen testing for detection of Legionella spp.: a systematic review. J Clin Microbiol.

[12] Mykietiuk A, Carratala J, Fernandez-Sabe N (2005). Clinical outcomes for hospitalized patients with Legionella pneumonia in the antigenuria era: the influence of levofloxacin therapy. Clin Infect Dis.

[13] Cecchini J, Tuffet S, Sonneville R (2017). Antimicrobial strategy for severe community-acquired legionnaires' disease: a multicentre retrospective observational study. J Antimicrob Chemother.

[14] Garcia-Vidal C, Sanchez-Rodriguez I, Simonetti AF (2017). Levofloxacin versus azithromycin for treating legionella pneumonia: a propensity score analysis. Clin Microbiol Infect.

[15] Jasper AS, Musuuza JS, Tischendorf JS, Stevens VW, Gamage SD, Osman F, Safdar N (2021). Are fluoroquinolones or macrolides better for treating Legionella pneumonia? A systematic review and meta-analysis. Clin Infect Dis.

[16] Kato H, Hagihara M, Asai N, Shibata Y, Koizumi Y, Yamagishi Y, Mikamo H (2021). Meta-analysis of fluoroquinolones versus macrolides for treatment of Legionella pneumonia. J Infect Chemother.

